# Engagement Patterns With #NaturalRemedies on TikTok: Formative Analysis

**DOI:** 10.2196/83274

**Published:** 2026-03-25

**Authors:** Apeksha Mewani, Corey H Basch, Keanu Renne-Glover, Vincent Jones II, Cristiana Cordero, Adea Gutiqi

**Affiliations:** 1 Health Equity, Administration, and Technology (HEAT), Lehman College, 250 Bedford Park Blvd W, Bronx, NY, 10468, United States, 1-718-960-2467; 2 Department of Public Health, William Paterson University of New Jersey, Wayne, NJ, United States; 3 Department of Health Sciences, NYC College of Technology, Brooklyn, NY, United States; 4 Department of Health and Human Performance, York College, Jamaica, NY, United States; 5 Lehman College, Bronx, NY, United States; 6 City University of New York, 365 5th Ave, New York, NY, 10016, United States, 1 9178585242

**Keywords:** natural remedies, TikTok, public health, alternative and integrative medicine, wellness industry, social media, influencers, health education, medical misinformation, patient trust, chronic illness, symptom management, self-care

## Abstract

This research letter provides formative evidence from a cross-sectional analysis of 118 TikTok videos under #NaturalRemedies, identifying content formats and expertise cues associated with higher engagement, while also demonstrating the feasibility of using engagement metrics and thematic coding to analyze health narratives on emerging platforms such as TikTok.

## Introduction

Social media platforms like TikTok are increasingly popular sources for public-health information and can shape health perceptions, decisions, and behaviors, especially via influencer-led health messaging [1]. TikTok’s short-form, hyper-specific algorithmic design accelerates the dissemination of wellness content, raising concerns about creator credibility and misinformation [2,3]. Health content on TikTok and related platforms shows patterns in education-oriented videos and influencer and audience dynamics and often lacks evidence citation [4-6]. Mental health and COVID-19–related content on TikTok varies in credibility, health messaging strategies, and user engagement patterns [7-9]. Our formative analysis identified themes linked to engagement, informing future digital public-health interventions in #NaturalRemedies videos. Such videos represent a distinct subset of wellness content characterized by home-based practices, herbal or dietary interventions, and limited regulatory oversight, raising concerns about evidence quality, credibility, and potential misinformation [3,5,8]. A formative analysis can explore engagement patterns early on, with descriptive studies offering the opportunity to generate hypotheses and inform future digital health communication strategies.

## Methods

### Overview

On February 21, 2025, we captured the 120 most widely viewed content videos under the hashtag #NaturalRemedies after sorting by view count. Videos that were not in English (and missing subtitles), missing audio, duplicates, or misclassified were excluded, leaving 118. Sampling was conducted on one day to capture a consistent cross-section of #NaturalRemedies content. The specific date was selected based on the availability of the data collector. The sample size was consistent with prior TikTok content analyses [4,6] reporting that 120 videos was feasible for analysis; this aligns with typical TikTok content-analysis studies that examine the 100 to 200 top-ranked posts [4-7]. For each video, we extracted likes, comments, shares, and saves, coded sentiment (positive, neutral, negative), formats (tutorial, narrative), creator expertise (credentialed expert, scientist or medical professional, influencer), and themes (educational content, DIY, gut and skin health). These codes were drawn from prior content-analysis literature [5]; the full codebook is provided in Multimedia Appendix 1. A 10% subsample was double-coded (κ=1) for all variables. No discrepancies were reported.

Descriptive statistics summarized engagement, Mann-Whitney *U* tests compared theme-present versus theme-absent groups, and Spearman correlations explored associations. Analyses were conducted in R (version 2024.12.0+467; R Foundation for Statistical Computing). Because engagement data were nonnormally distributed (Shapiro-Wilk *P*<.05), nonparametric tests were used with a 2-tailed α=.05. These methods align with prior social media health content analyses [4,6,10].

### Ethical Considerations

Institutional review board approval was deemed unnecessary as the study did not involve human subjects.

## Results

Descriptive statistics are summarized in Table S1 in Multimedia Appendix 1; inferential comparisons appear in Table S2 in Multimedia Appendix 1. Positive sentiment predominated (98/118) and corresponded with higher medians for likes and saves. Tutorials were associated with greater sharing and saving (shares: median 8310, IQR 3692-18,200 vs 5661, IQR 1981-10,100; *U*=1194.5; *P*=.04; saves: median 15,600, IQR 7795-30,900 vs 11,700, IQR 3578-21,500; *U*=1204.5; *P*=.04). Videos without DIY content received more likes (median 51,150, IQR 36,975-73,150 vs 35,150, IQR 18,775-64,87; *U*=1070.5; *P*=.046) and comments (median 973.5, IQR 429.5-1557 vs 469.5, IQR 157.25-907.5; *U*=1152.5; *P*=.008) than DIY videos.

Credentialed-expert videos (Σ_n_=118; n=2) achieved the highest engagement (likes: median 120,200, IQR 100,050-140,350; *U*=15.5; *P*=.04), compared to the median like count of 39,000 (IQR 21,075-65,025) for nonexpert-created videos. Despite content categorized under “credentialed experts” representing approximately 1.69% (Σ_n_=118; n=2) of the sample, 93.2% (Σ_n_=118; n=110) of videos were coded as “educational content,” and videos were marked as “not present” for “myth busting.”

“Connection with nature” produced more comments when present (median 1321, IQR 744-1521 vs 477, IQR 184-909; *U*=239.5; *P*=.01). “Gut health” content showed higher shares when present (median 12,200, IQR 6211.5-20,750 vs 6106.5, IQR 391.75-13,950; *U*=1076.5; *P*=.02) and trended toward more comments and saves (*P*=.07 and *P*=.06), although this was not significant. Educational content approached significance for likes (*P*=.10). Personal-experience narratives differed in shares (*P*=.03), with lower medians when present in this sample. Furthermore, median like count for videos with the intent to sell a product was almost equivalent to those without such intent (respectively, 39,800, IQR 28,100-51,300 vs 39,900, IQR 20,850-66,750), despite the uneven distribution of videos with and without commercial intent (Σ_n_=118; n=11 vs n=107).

## Discussion

This formative analysis showed that engagement with natural-remedy content was shaped by sentiment, format, and expertise cues. Tutorials use a practical, action-oriented format, which was consistently associated with higher shares and saves, while DIY content underperformed. Rare appearances of credentialed experts corresponded with notably higher engagement, suggesting meaningful audience responsiveness to professional authority. Given that influencers and wellness enthusiasts produced almost all videos, health professionals thus have an important opportunity to increase their visibility in natural-remedy discussions on TikTok. Because natural remedies are often informal, experiential, and weakly regulated, understanding engagement patterns is especially relevant for assessing how health claims circulate on social media.

Our findings complement prior reports on influencer dynamics, education-oriented health videos and their credibility, and platform-specific engagement patterns [4,6,8,9]. Recent TikTok research has shown that users are highly responsive to content that is framed in an educational, structured, or professionally anchored manner, including studies of mental-health messaging and COVID-19 communications [7,8]. These results extend prior work by showing that similar engagement patterns apply to natural-remedy content, an area still under-studied despite its rapid growth. Elevated engagement with gut health and nature-linked themes reflects broader wellness trends across TikTok. The lack of myth-busting content, amid abundant educational videos, highlights persistent gaps in corrective, evidence-based messaging, mirroring earlier findings on wellness influencers, where evidence of citation and scientific grounding is often limited [1,3].

Tutorials requiring minimal user effort outperformed DIY formats, suggesting usability drives engagement. Expert-led or medically aligned videos also performed strongly, indicating viewer preference for legitimacy cues, mirroring findings from EduTok, naturopathic influencer behavior, and TikTok nutrition studies, which likewise emphasize credibility, structure, and clear visual demonstration [4,6,9].

Study limitations include the cross-sectional design, single-hashtag sampling, and absence of demographic data. Results from one day’s algorithmic rankings may shift with platform trends and changing exposure patterns. View-count sorting may favor algorithmically boosted videos, though it captures the content most visible to users.

Overall, these results illustrate the feasibility of using social-media analytics to guide digital public-health communication and intervention design [10] and underscore the value of formative work in identifying emerging communication patterns. Given strong engagement with tutorials, expert-led messages, and wellness themes such as gut health, public-health practitioners may consider leveraging TikTok to disseminate evidence-based guidance. Future research should test messaging strategies, explore content accuracy, and examine how platform algorithms shape exposure to expert versus nonexpert information.

## Supplementary material

10.2196/83274 Multimedia Appendix 1 TikTok video differences in the median numbers of likes, comments, shares, and saves by theme, with descriptive statistics.
